# Method validation of a set of 12 GEM® Premier™ 4000 blood gas analyzers for point-of-care testing in a university teaching hospital

**DOI:** 10.1016/j.plabm.2017.12.001

**Published:** 2017-12-13

**Authors:** Charlotte Oris, Yoan Clavel, Matthieu Jabaudon, Annick Pialat, Hadj Abdelkader Mohamed, Frédérique Lioret, Vincent Sapin, Damien Bouvier

**Affiliations:** a Clermont-Ferrand Teaching Hospital, Biochemistry and Molecular Biology Department, F63000 Clermont-Ferrand, France; b Clermont-Ferrand Teaching Hospital, Intensive Care Unit, F63000 Clermont-Ferrand, France; c Université Clermont Auvergne, CNRS, Inserm, GReD, F-63000 Clermont-Ferrand, France; d Clermont-Ferrand Teaching Hospital, Hemodialysis and Nephrology Unit, F63000 Clermont-Ferrand, France

**Keywords:** Blood gas, Point-of-care testing, Method validation, NF ISO 15189, NF ISO 22870

## Abstract

**Background:**

Blood gas analyzers are o0.ften integrated into point-of-care testing provisions. International standards (ISO 22870 and 15189) as adapted to French COFRAC regulations make accreditation of point-ofta-care testintag obligatory. We installed and assessed 12 GEM PREMIER 4000 analyzers for pH, *p*CO_2_, *p*O_2_, Na^+^, K^+^, Cl^-^, Ca^2+^, lactate, hemoglobin and oxyhemoglobin (O_2_Hb) at Clermont-Ferrand Hospital. These instruments were distributed across 11 care sites in the hospital.

**Methods:**

Precision was studied at two control levels for each parameter. Comparisons between GEM analyzers were performed (on 30 samples) for pH, *p*CO_2_, *p*O_2_, Na^+^, K^+^, Cl^-^, Ca^2+^, lactate, hemoglobin and O_2_Hb; and between GEM analyzers and the central laboratory for Na^+^, K^+^, Cl^-^, Ca^2+^ and hemoglobin (on 30–50 samples). Uncertainty in measurement (UM) was evaluated with an approach using reproducibility and accuracy data.

**Results:**

The coefficients of variation (CVs) were in line with recommendations, except for the repeatability CV for *p*O_2_. All CVs were below 4%. All comparisons complied with recommendations. Uncertainties of measurement were also validated.

**Conclusion:**

Our results met standard requirements and the 12 analyzers were assessed as suitable for point-of-care testing in services of academic medical centers, as exemplified at Clermont-Ferrand hospital.

## Highlights

• Complete method validation of 12 analyzers for POCT in a multisite hospital.
• First published values of uncertainty in measurement very useful for pO2 and O_2_Hb.
• Comparison between GEM4000 and central lab analyzers for Na^+^, Cl^-^, K^+^, lactate, Hb.

## 1 Introduction

In France, all biomedical analysis laboratories are governed by the standardization and accreditation regulations set by COFRAC to meet the requirements of the standard EN ISO 15189. However, point-of-care testing (POCT), governed by the standard EN ISO 22870, also requires accreditation by 2020. Although biomedical laboratories have moved forward in the accreditation of analyses carried out in the laboratory, currently very few of them are accredited for POCT. Although Normes Françaises (NF) 22870 is an extension of 15189, and shares many of its requirements, there are some additional requirements specific for POCT. For example, an oversight committee must be created and established prior to installing POCT equipment [1], [2]. The training and accreditation of the users, both clinical and laboratory staff, are also essential steps, and must be traceable [3], [4]. A hospital biologist oversees POCT [1], [5], [6], [7].

We note that POCT is clearly defined as “an analysis carried out close to the patient or to the place where the patient is, the result of which may lead to a modification of the care given to the patient” [5], [8]. Several aspects of pH and blood gas analysis are unique in laboratory analysis, and furthermore, no other test results have such a great and immediate impact on patient care [9]. In addition, it is now possible to carry out further vital biological assays on the same sample, e.g. of ions, lactate, hemoglobin, and ionized calcium, and to measure co-oximetry values. These results provide an overall picture of the patient's state of health, which is especially important for patients admitted to adult and neonatal resuscitation wards (scalp pH), or in emergency medicine [10], [11]. Ancy et al. report that more and more hospitals recognize the value of broadening the range of measurements obtained from automated blood gas analyzers [12]. In addition to these vital aspects, POCT is fundamentally useful, particularly for blood gases, in that it reduces pre-analytical errors, which alone account for some 62% of medical errors [13], [14], [15]. Rapid, reliable, easy-to-maintain cassette analyzers are currently available which are ideally suited to this kind of testing for the user (physicians, nurses and midwives). Furthermore, it has been demonstrated that qualified nurses spend only 37% of their working time with patients [16]. Hence this type of analyzer offers an alternative solution with many advantages, especially that of favoring closer patient care by reducing avoidable workload. Due to the closer proximity of POCT analyzers, the turnaround time for blood gases can be improved.

At the Clermont-Ferrand university teaching hospital, as part of a transfer of activity to the medical biochemistry laboratory, the blood gas analysis equipment was upgraded. For this purpose, Werfen equipment was selected, particularly for its analytical performance and ease of use for both clinical and laboratory staff; but most importantly, for the supplier's fully independent iQM system [6], [7], [13], [17], [18]. In all, 12 GEM PREMIER 4000 analyzers were deployed across 11 sites, throughout two hospitals, CHU Gabriel Montpied and CHU Estaing. The method validation process for the 12 devices was intricate and complex, due to the disparity between the two hospital centers. The classical steps for appraising standard-compliance on-site, specifically precision studies and method comparisons, are necessary for the validation of the analytical procedure according to the standard EN ISO 22870. Our aim was to validate the methods for the 12 analyzers installed.

## 2 Materials and methods

### 2.1 Analyzers

**Fig. 1:**
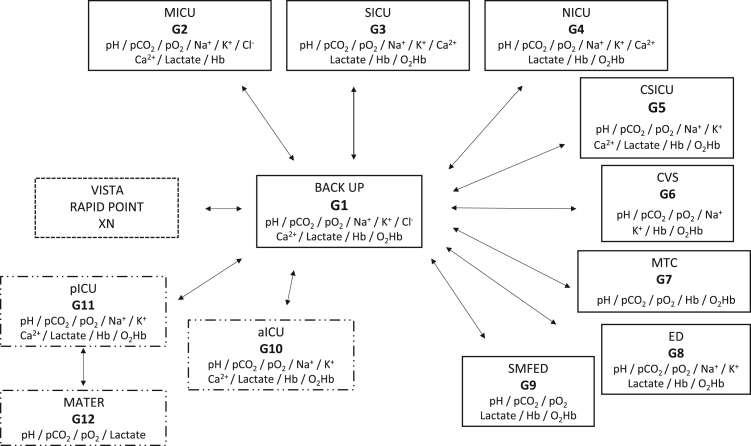
Diagram showing comparisons made between 12 GEM PREMIER 4000 analyzers (G1–G12), and between G1 and the central laboratory analyzers. pH: hydrogen potential, *p*CO_2_: partial pressure of CO_2_, *p*O_2_: partial pressure of O_2_, Na^+^: sodium, K^+^: potassium, Cl^-^: chloride, Ca^2+^: calcium, Hb: hemoglobin, O_2_Hb: oxyhemoglobin. MICU: medical intensive care unit, SICU: surgical intensive care unit, NICU: neurological intensive care unit, CSICU: cardiovascular surgery intensive care unit, CVS: cardiovascular surgery operating theater, MTC: medico-technical center, ED: emergency department, SMFED: sport medicine and functional exploration department, aICU: adult intensive care unit, pICU: pediatric intensive care unit, MATER: maternity ward.- - - - - - - - - Central Laboratory **————** Gabriel Montpied Hospital — - - — - - — Estaing Hospital.

The GEM PREMIER 4000 analyzers (Werfen, Le Pré-Saint-Gervais, France) are cassette blood gas analysis instruments. Proton concentration [H+] (for Hydrogen potential (pH)), partial pressure of CO_2_ (*p*CO_2_), sodium (Na^+^), potassium (K^+^), chloride (Cl^-^) and ionized calcium (Ca^2+^) levels are measured by potentiometry; partial pressure of O_2_ (*p*O_2_) and lactate are measured by amperometry; hemoglobin (Hb) and oxyhemoglobin (O_2_Hb) by optical absorbance. Twelve analyzers were deployed over 11 sites of the Clermont-Ferrand university teaching hospital system between February and June 2015. At these 11 deployment sites, each analyzer was assigned a name according to the clinical ward where it was located (Fig. 1). At Gabriel Montpied hospital, the instruments were located in the medical intensive care unit (MICU or G2), surgical intensive care unit (SICU or G3), neurological intensive care unit (NICU or G4), cardiovascular surgery intensive care unit (CSICU or G5), cardiovascular surgery operating theater (CVS or G6), medico-technical center (MTC or G7), emergency department (ED or G8), and sport medicine and functional exploration department (SMFED or G9). A backup instrument (BACKUP or G1) was also installed at the SICU. At Estaing hospital, the instruments were installed in the adult intensive care unit (aICU or G10), pediatric intensive care unit (pICU or G11) and maternity ward (MATER or G12). To allow for different specific clinical requirements, not all the analytical values were available on all the analyzers (Fig. 1).

### 2.2 Repeatability

Tests to evaluate repeatability were carried out using vials of two distinct levels of controls supplied by Werfen. These were titered aqueous solutions, with physiological analyte concentrations for level 2 and pathological concentrations for level 3, designated respectively GEM System Evaluator 2 (GSE2) and 3 (GSE3). For all the instruments, 30 vials of GEM System Evaluator 2 (Reference no. 00025000102) and 30 vials of GEM System Evaluator 3 (Reference no. 00025000103) were analyzed. The mean of the expected values for our different parameters in solutions GSE2 and GSE3 are presented in Table 1.

**Table 1:** Supplier's target values for GEM System Evaluator solutions 2 (GSE2) and 3 (GSE3), and solutions A, B and D carried on iQM® cartridges in GEM PREMIER 4000 analyzers.

	**Unit**	**GSE 2**	**GSE 3**	**Solution A**	**Solution B**	**Solution D**
**pH**	–	7.38	7.57	6.91	7.40	–
**[H**^**+**^**]**	nmol/L	41.7	26.9	123.0	39.8	–
***p*****CO**_**2**_	mmHg	34	14	65.5	33.4	–
***p*****O**_**2**_	mmHg	88	365	112	178	–
**Na**^**+**^	mmol/L	141	155	107	153	–
**K**^**+**^	mmol/L	4.6	7.6	7.0	2.0	–
**Cl**^**-**^	mmol/L	107	140	47	88.5	–
**Ca**^**2+**^	mmol/L	1.14	0.65	1.74	0.79	–
**Lactate**	mmol/L	0.80	2.40	3.27	–	8.17
**Hb**	g/dL	14.6	7.6	14.5	–	7.3
**O**_**2**_**Hb**	%	73.1	92.9	89.4	–	69.8

pH: hydrogen potential, [H^+^]: proton concentration, *p*CO_2_: partial pressure of CO_2_, *p*O_2_: partial pressure of O_2_, Na^+^: sodium, K^+^: potassium, Cl^-^: chloride, Ca^2+^: calcium, Hb: hemoglobin, O_2_Hb: oxyhemoglobin.

### 2.3 Reproducibility

The reproducibility of the GEM PREMIER 4000 analyzers was assessed independently as soon as the cartridge was loaded (6)(17)(18). Three internal quality control solutions (A B and D) were programmed for measurements at regular time intervals. For [H^+^], *p*CO_2_, *p*O_2_, Na^+^, K^+^, Ca^2+^ and Cl^-^, assays were carried out on solutions A and B. The assays of lactate, hemoglobin (Hb) and oxyhemoglobin (O_2_Hb) were carried out on solutions A and D. The data were then retrieved and processed using the PCSar software supplied by Werfen (V2.0). The means of the expected values for the various parameters in solutions A, B and D are presented in Table 1.

### 2.4 Accuracy

The GEM PREMIER 4000 analyzers were enrolled in an external quality assessment (EQA) program from ASQUALAB association (Paris, France). Accuracy is evaluated from EQA result with the percentage bias calculated as (100*(((laboratory result)-(expected result))/(expected result)). For each parameter and at 2 levels (similar to those studied for reproducibility), an average of 3 bias determinations was calculated.

### 2.5 Uncertainty in measurement

Uncertainty in measurement (UM) was evaluated with an approach using both reproducibility and accuracy data. For each parameter and at 2 levels (the same as those studied for reproducibility), results were calculated for concentrations and as a percentage.

$$\mathrm{UM}=2*\surd \left({\left(\mathrm{reproducibility}\;\mathrm{SD}\right)}^{2}+{\left(\mathrm{uncertainty}\;\mathrm{from}\;\mathrm{accuracy}\right)}^{2}\right)$$

$$\mathrm{Uncertainty}\;\mathrm{from}\;\mathrm{accuracy}=\surd ({\left(D/\surd 3\right)}^{2}+\sigma_{D}^{2})$$

SD: Standard Deviation; D: gap average = (Σe)/n; e: laboratory result – reference mean (peer group); σ_D_: gap standard deviation

### 2.6 Method comparison

The comparisons made between analyzers are shown in Fig. 1. First, so as to serve as the reference analyzer, BACKUP (G1) was compared with the automated analyzers of the central laboratory. Na^+^, K^+^, Cl^-^ and lactate were assayed at the Medical Biochemistry and Molecular Biology Laboratory on a Vista®-type analyzer (Siemens, Saint-Denis, France). Ca^2+^ was assayed in the same laboratory on a RAPIDPoint500® analyzer (Siemens, Saint-Denis, France). Hb was assayed on an XN® analyzer (Sysmex, Villepinte, France) in the Hematology Laboratory. pH, *p*O_2_, *p*CO_2_ and O_2_Hb were only determined as point-of-care testing in the clinical departments at the Clermont-Ferrand university teaching hospital. All the analyzers were then compared with G1, except for the one in the maternity ward (G12), which was compared with G11 (Fig. 1). For each comparison, the time interval between the two assays was negligible because analyzers were first installed side by side. The comparisons between GEM PREMIER 4000 analyzers were made using 30 samples (35 for Ca^2+^). The comparisons between G1 and the central laboratory were made using 50 samples for Na^+^ and Cl^-^, 40 samples for K^+^ and lactate, and 30 samples for Ca^2+^. For the comparison between the central laboratory and G1, samples of whole blood were taken with a heparinized syringe (for analysis on G1) and plasma from the same blood sample was taken for the central laboratory analysis. For the comparisons between GEM PREMIER 4000 analyzers, samples in heparinized syringes were collected from SICU, MICU and NICU. All the samples were anonymized. To obtain a broader range of results for several of the parameters such as *p*O_2_, *p*CO_2_, lactates and O_2_Hb, some samples were left to age at room temperature. In parallel, jointly with the hemodialysis and cardiovascular surgery intensive care units, we collected samples from pre- and post-dialysis patients to obtain extreme values of K^+^ and low values of Ca^2+^ (post-filtered samples from extra-renal clearance with citrate [19]). Lastly, jointly with the NICU, we collected samples from patients under osmotherapy to obtain extreme values of Na^+^ [20]. We followed the French Society of Clinical Biology (SFBC) guidelines for the spread of points over the range for each parameter [21].

A second method comparison between the 12 GEM PREMIER 4000 analyzers was made using GEM System Evaluator vials. The comparisons between GEM PREMIER 4000 analyzers were made on 43 vials (5 of level 1, 19 of level 2 and 19 of level 3), all from the same batch (batch 1512 for level 1, 2513 for level 2 and 3514 for level 3), run on all 12 analyzers.

### 2.7 Statistics

The data was analyzed using the VISKALI® software (Viskali ACC, Lyon, France, V5.0). For the accuracy study, we calculated means (± SD) of repeatability and reproducibility, together with coefficients of variation (CV) expressed as a percentage. The acceptability criteria chosen were either the desirable specifications of Ricos based on biological variation, in particular for hemoglobin in repeatability and reproducibility tests, and also K^+^ for reproducibility [22], or the specifications of the French Society of Clinical Biology (SFBC) for all the other repeatability and reproducibility tests as well as accuracy tests [21]. The method comparison was studied by least rectangles regression and calculation of means (+ SD) of differences and ratios. The corresponding graphs were plotted. The comparison of the means was obtained after analysis of the 43 GEM System Evaluator vials on each of the 12 analyzers, and were made with an ANOVA test. Statistical significance of differences was set at p < 0.05.

## 3 Results

### 3.1 Repeatability

The results obtained for the determination of [H^+^], *p*CO_2_, Na^+^, Cl^-^, K^+^, Ca^2+^, lactate and O_2_Hb all complied with SFBC recommendations, with corresponding CV values below 1.5%, 3.8%, 0.8%, 1.2%, 1.2%, 1.2%, 3.8% and 3.8% respectively (Table 2). Hb was compliant on all the analyzers, but for the desirable specifications of Ricos et al. [22] with CV values below 1.07% (Table 2). However, for *p*O_2_, we observed that only three values of CV obtained met SFBC requirements: level 3 for analyzer G2; and levels 2 and 3 for G12 (Table 2). CV values for repeatability all conformed to the supplier's standards.

**Table 2:** Repeatability studies on 12 GEM PREMIER 4000 analyzers (G1–G12) at 2 levels. The results are presented as coefficients of variation (%). Thirty vials of GEM System Evaluator 2 and 30 vials of GEM System Evaluator 3 were analyzed.

	**[H**^**+**^**]**		***p*****CO**_**2**_		***p*****O**_**2**_		**Na**^**+**^		**Cl**^**-**^		**K**^**+**^		**Ca**^**2+**^		**Lactate**		**Hb**		**O**_**2**_**Hb**	
	**Lev 2**	**Lev 3**	**Lev 2**	**Lev 3**	**Lev 2**	**Lev 3**	**Lev 2**	**Lev 3**	**Lev 2**	**Lev 3**	**Lev 2**	**Lev 3**	**Lev 2**	**Lev 3**	**Lev 2**	**Lev 3**	**Lev 2**	**Lev 3**	**Lev 2**	**Lev 3**
**G1**	0.94^a^^b^	0.86^a^^b^	1.46^a^^b^	2.56^a^^b^	2.00^b^	2.61^b^	0.69^a^^b^	0.83^b^	0.59^a^^b^	0.62^a^^b^	1.08^a^^b^	0.74^a^^b^	1.08^a^^b^	1.18^a^^b^	0.00^a^^b^	0.76^a^^b^	0.41^c^^b^	0.61^c^^b^	0.00^a^^b^	0.02^a^^b^
**G2**	1.17^a^^b^	1.00^a^^b^	1.60^a^^b^	2.68^a^^b^	1.90^b^	1.05^a^^b^	0.47^a^^b^	0.39^a^^b^	0.47^a^^b^	0.25^a^^b^	1.04^a^^b^	0.65^a^^b^	0.84^a^^b^	0.98^a^^b^	0.00^a^^b^	1.06^a^^b^	0.69^c^^b^	0.98^c,b^	–	–
**G3**	0.87^a^^b^	0.43^a^^b^	1.72^a^^b^	3.26^a^^b^	3.22^b^	3.77^b^	0.41^a^^b^	0.47^a^^b^	–	–	0.40^a^^b^	0.40^a^^b^	0.66^a^^b^	0.85^a^^b^	1.97^a^^b^	3.20^a^^b^	0.48^c^^b^	0.65^c^^b^	0.00^a^^b^	0.02^a^^b^
**G4**	0.42^a^^b^	0.43^a^^b^	1.25^a^^b^	2.28^a^^b^	3.47^b^	2.03^b^	0.38^a^^b^	0.62^a^^b^	–	–	0.57^a^^b^	0.78^a^^b^	0.71^a^^b^	1.15^a^^b^	0.00^a^^b^	1.68^a^^b^	0.47^c^^b^	0.34^c^^b^	0.00^a^^b^	0.02^a^^b^
**G5**	0.00^a^^b^	1.15^a^^b^	1.53^a^^b^	1.31^a^^b^	1.80^b^	2.14^b^	0.57^a^^b^	0.63^a^^b^	–	–	0.93^a^^b^	0.69^a^^b^	1.00^a^^b^	1.13^a^^b^	0.00^a^^b^	1.73^a^^b^	0.48^c^^b^	0.34^c^^b^	0.00^a^^b^	0.02^a^^b^
**G6**	0.00^a^^b^	0.02^a^^b^	1.59^a^^b^	2.95^a^^b^	2.18^b^	1.88^b^	0.58^a^^b^	0.58^a^^b^	–	–	0.70^a^^b^	0.61^a^^b^	–	–	–	–	0.64^c^^b^	0.54^c^^b^	0.00^a^^b^	0.00^a^^b^
**G7**	1.19^a^^b^	1.17^a^^b^	1.45^a^^b^	2.33^a^^b^	2.25^b^	2.49^b^	–	–	–	–	–	–	–	–	–	–	0.00^c^^b^	1.33^c^^b^	0.03^a^^b^	0.02^a^^b^
**G8**	0.71^a^^b^	1.28^a^^b^	0.99^a^^b^	2.49^a^^b^	2.19^b^	2.18^b^	0.58^a^^b^	0.93^b^	–	–	0.80^a^^b^	1.20^a^^b^	–	–	3.86^a^^b^	2.15^a^^b^	0.44^c^^b^	0.98^c,b^	0.00^a^^b^	0.02^a^^b^
**G9**	0.70^a^^b^	1.17^a^^b^	1.80^a^^b^	2.05^a^^b^	2.28^b^	1.63^b^	–	/	–	–	–	–	–	–	0.00^a^^b^	1.06^a^^b^	0.46^c^^b^	0.34^c^^b^	0.00^a^^b^	0.02^a^^b^
**G10**	1.10^a^^b^	0.43^a^^b^	1.66^a^^b^	3.35^a^^b^	2.09^b^	1.67^b^	0.32^a^^b^	0.35^a^^b^	–	–	0.55^a^^b^	0.46^a^^b^	0.66^a^^b^	0.85^a^^b^	0.00^a^^b^	2.00^a^^b^	0.41^c^^b^	0.63^c^^b^	0.06^a^^b^	0.04^a^^b^
**G11**	0.87^a^^b^	0.59^a^^b^	2.30^a^^b^	2.56^a^^b^	3.49^b^	3.04^b^	0.29^a^^b^	0.37^a^^b^	–	–	0.55^a^^b^	0.76^a^^b^	0.67^a^^b^	0.93^a^^b^	3.20^a^^b^	1.45^a^^b^	0.32^c^^b^	0.65^c^^b^	0.06^a^^b^	0.04^a^^b^
**G12**	0.87^a^^b^	1.17^a^^b^	0.02^a^^b^	0.03^a^^b^	0.01^a^^b^	0.03^a^^b^	–	–	–	–	–	–	–	–	0.06^a^^b^	0.02^a^^b^	–	–	–	–
***Mean***	***0.74***	***0.81***	***1.45***	***2.32***	***2.46***	***2.34***	***0.54***	***0.58***	***0.53***	***0.43***	***0.82***	***0.70***	***0.80***	***1.07***	***0.91***	***1.51***	***0.44***	***0.67***	***0.02***	***0.02***
																				
***SFBC specifications***	***1.5***	***1.5***	***3.8***	***4.5***	***1.5***	***1.5***	***0.8***	***0.7***	***1.2***	***1.2***	***1.2***	***1.2***	***1.2***	***1.2***	***3.8***	***3.8***	***1.07***^c^	***1.07***^c^	***3.8***	***3.8***
***Manufacturer specifications***	***2***	***2***	***7.1***	***10***	***5.3***	***4.6***	***1.8***	***1.9***	***2.4***	***1.9***	***4.4***	***3.3***	***3.9***	***6.1***	***17***	***10***	***2.8***	***4.7***	***1.3***	***1.1***

[H^+^]: proton concentration, *p*CO_2_: partial pressure of CO_2_, *p*O_2_: partial pressure of O_2_, Na^+^: sodium, K^+^: potassium, Cl^-^: chloride, Ca^2+^: calcium, Hb: hemoglobin, O_2_Hb: Oxyhemoglobin.

a Result meets SFBC specifications [21].

b Result meets manufacturer's specifications.

c Result meets desirable specifications of Ricos et al. [22] after conversion of a reproducibility objective into a repeatability objective according to SFBC recommendations [21] (application of the formula CV repeatability = CV reproducibility * 0.75).

### 3.2 Reproducibility

The CV values for reproducibility obtained met SFBC requirements for [H^+^], *p*O_2_, *p*CO_2_, Na^+^, K^+^, Cl^-^, Ca^2+^, lactate and O_2_Hb with CV values respectively below 2%, 2%, 5%, 1.1%, 1.6%, 1.6%, 1.6%, 5% and 5%. Lastly, the CV values obtained for Hb complied with the recommendations of Ricos et al. (1.43%) (Table 3).

**Table 3:** Reproducibility study on the 12 GEM PREMIER 4000 analyzers (G1–G12) on two levels. The results are presented as coefficients of variation in percentage. The reproducibility is evaluated independently as soon as the cartridge is loaded. Three internal quality control solutions were programmed to be measured at regular time intervals. For [H^+^], *p*CO_2_, *p*O_2_, Na^+^, K^+^, Ca^2+^ and Cl^-^, assays were carried out on solutions A and B; for lactate, Hb and O_2_Hb, on solutions A and D.

	**[H**^**+**^**]**		***p*****CO**_**2**_		***p*****O**_**2**_		**Na**^**+**^		**Cl**^**-**^		**K**^**+**^		**Ca**^**2+**^		**Lactate**		**Hb**		**O**_**2**_**Hb**	
	**Sol A**	**Sol B**	**Sol A**	**Sol B**	**Sol A**	**Sol B**	**Sol A**	**Sol B**	**Sol A**	**Sol B**	**Sol A**	**Sol B**	**Sol A**	**Sol B**	**Sol A**	**Sol D**	**Sol A**	**Sol D**	**Sol A**	**Sol D**
**G1**	0.49^a^^b^	1.47^a^^b^	1.08^a^^b^	2.73^a^^b^	1.20^a^^b^	1.77^a^^b^	0.40^a^^b^	0.74^a^^b^	0.00^a^^b^	0.75^a^^b^	0.39^a^^b^	2.12^c^^b^	0.69^a^^b^	1.14^a^^b^	1.80^a^^b^	0.86^a^^b^	0.46^c^^b^	0.00^c^^b^	0.00^a^^b^	0.09^a^^b^
**G2**	0.80^a^^b^	1.97^a^^b^	0.94^a^^b^	2.76^a^^b^	1.20^a^^b^	1.99^a^^b^	0.45^b^	0.62^a^^b^	0.00^a^^b^	0.84^a^^b^	0.71^a^^b^	0.20^a^^b^	0.63^a^^b^	1.12^a^^b^	1.81^a^^b^	2.39^a^^b^	0.25^c^^b^	0.40^c^^b^	–	–
**G3**	0.66^a^^b^	1.87^a^^b^	1.07^a^^b^	2.74^a^^b^	1.00^a^^b^	1.29^a^^b^	0.38^a^^b^	0.77^a^^b^	–	–	0.75^a^^b^	0.35^a^^b^	0.51^a^^b^	1.00^a^^b^	1.51^a^^b^	1.21^a^^b^	0.38^c^^b^	0.00^c^^b^	0.00^a^^b^	0.09^a^^b^
**G4**	0.46^a^^b^	1.56^a^^b^	1.15^a^^b^	1.40^a^^b^	1.16^a^^b^	1.59^a^^b^	0.30^a^^b^	0.66^a^^b^	–	–	0.43^a^^b^	2.12^c^^b^	0.51^a^^b^	1.14^a^^b^	1.80^a^^b^	1.38^a^^b^	0.34^c^^b^	0.00^c^^b^	0.00^a^^b^	0.09^a^^b^
**G5**	0.82^a^^b^	1.65^a^^b^	1.05^a^^b^	2.44^a^^b^	1.36^a^^b^	1.34^a^^b^	0.54^a^^b^	0.79^a^^b^	–	–	0.71^a^^b^	0.30^a^^b^	0.63^a^^b^	1.37^a^^b^	1.81^a^^b^	2.79^a^^b^	0.44^c^^b^	0.72^c^^b^	0.00^a^^b^	0.26^a^^b^
**G6**	0.59^a^^b^	1.31^a^^b^	0.83^a^^b^	1.36^a^^b^	1.47^a^^b^	1.32^a^^b^	0.46^a^^b^	0.55^a^^b^	–	–	0.48^a^^b^	1.73^a^^b^	–	–	–	–	0.50^c^^b^	0.49^c^^b^	0.00^a^^b^	0.12^a^^b^
**G7**	0.62^a^^b^	1.25^a^^b^	1.51^a^^b^	2.34^a^^b^	0.87^a^^b^	1.60^a^^b^	–	–	–	–	–	–	–	–	–	–	0.33^c^^b^	0.19^c^^b^	0.00^a^^b^	0.10^a^^b^
**G8**	0.55^a^^b^	1.64^a^^b^	0.70^a^^b^	2.78^a^^b^	1.34^a^^b^	1.61^a^^b^	0.25^a^^b^	0.61^a^^b^	–	–	0.69^a^^b^	0.30^a^^b^	–	–	1.81^a^^b^	0.87^a^^b^	0.46^c^^b^	0.00^c^^b^	0.00^a^^b^	0.10^a^^b^
**G9**	0.40^a^^b^	1.30^a^^b^	1.17^a^^b^	2.33^a^^b^	1.35^a^^b^	1.61^a^^b^	–	–	–	–	–	–	–	–	1.80^a^^b^	1.23^a^^b^	0.37^c^^b^	0.18^c^^b^	0.01^a^^b^	0.12^a^^b^
**G10**	1.01^a^^b^	1.53^a^^b^	0.94^a^^b^	2.29^a^^b^	0.96^a^^b^	1.51^a^^b^	0.58^a^^b^	0.73^a^^b^	–	–	0.74^a^^b^	0.20^a^^b^	0.69^a^^b^	1.14^a^^b^	1.80^a^^b^	1.49^a^^b^	0.39^c^^b^	0.00^c^^b^	0.00^a^^b^	0.10^a^^b^
**G11**	0.62^a^^b^	1.65^a^^b^	1.48^a^^b^	2.47^a^^b^	1.24^a^^b^	1.60^a^^b^	0.49^a^^b^	0.68^a^^b^	–	–	0.37^a^^b^	2.24^c^^b^	0.57^a^^b^	1.00^a^^b^	1.80^a^^b^	1.61^a^^b^	0.31^c^^b^	0.45^c^^b^	0.00^a^^b^	0.07^a^^b^
**G12**	1.17^a^^b^	1.50^a^^b^	1.17^a^^b^	2.17^a^^b^	1.38^a^^b^	1.60^a^^b^	–	–	–	–	–	–	–	–	1.80^a^^b^	1.12^a^^b^	–	–	–	–
***Mean***	***0.68***	***1.56***	***1.09***	***2.32***	***1.21***	***1.57***	***0.43***	***0.68***	***0.00***	***0.80***	***0.59***	***1.1***	***0.60***	***1.13***	***1.77***	***1.50***	***0.38***	***0.22***	***0.00***	***0.11***
																				
***SFBC specifications***	***2***	***2***	***5***	***6***	***2***	***2***	***1.3***	***1.1***	***1.6***	***1.6***	***1.6***	***2***	***1.6***	***1.6***	***5***	***5***	–	–	***5***	***5***
***Ricos et al. specifications***	–	–	–	–	–	–	–	–	–	–	***2.3***	***2.3***	–	–	–	–	***1.43***	***1.43***	–	–
***Manufacturer specifications***	***2***	***2***	***5.1***	***7.1***	***5.3***	***4.6***	***2***	***1.9***	***2.9***	***2.4***	***3.3***	***2.4***	***3.2***	***6.1***	***10***	***5.4***	***2.8***	***4.7***	***1.1***	***1.3***

[H^+^]: proton concentration, *p*CO_2_: partial pressure of CO_2_, *p*O_2_: partial pressure of O_2_, Na^+^: sodium, K^+^: potassium, Cl^-^: chloride, Ca^2+^: calcium, Hb: hemoglobin, O_2_Hb: oxyhemoglobin.

a Result meets SFBC specifications [21].

b Result meets manufacturer's specifications.

c Result meets desirable specifications of Ricos et al. [22].

### 3.3 Accuracy

The bias values obtained met SFBC requirements for [H^+^], *p*O_2_, *p*CO_2_, Na^+^, K^+^, Cl^-^, Ca^2+^, lactate and O_2_Hb (Table 4). The bias values for Hb were considered as “good” by ASQUALAB.

**Table 4:** Accuracy study on the 12 GEM PREMIER 4000 analyzers (G1–G12) at two levels. The results are presented as an average of 3 bias determinations in percent. For [H^+^], *p*CO_2_, *p*O_2_, Na^+^, K^+^, Ca^2+^ and Cl^-^, calculations were based on sample concentrations similar to those of solutions A and B. For lactate, Hb and O_2_Hb, calculations were based on sample concentrations similar to those of solutions A and D.

	**[H**^**+**^**]**		***p*****CO**_**2**_		***p*****O**_**2**_		**Na**^**+**^		**Cl**^**-**^		**K**^**+**^		**Ca**^**2+**^		**Lactate**		**Hb**		**O**_**2**_**Hb**	
	**Sol A**	**Sol B**	**Sol A**	**Sol B**	**Sol A**	**Sol B**	**Sol A**	**Sol B**	**Sol A**	**Sol B**	**Sol A**	**Sol B**	**Sol A**	**Sol B**	**Sol A**	**Sol D**	**Sol A**	**Sol D**	**Sol A**	**Sol D**
**G1**	0.69^a^	2.16^a^	0.54^a^	0.49^a^	0.90^a^	1.60^a^	0.25^a^	0^a^	0.31^a^	0.85^a^	0.88^a^	2.27^a^	0.59^a^	0.76^a^	2.33^a^	2.86^a^	0.76	1.53	1.08^a^	1.35^a^
**G2**	1.62^a^	1.27^a^	1.06^a^	0.44^a^	1.10^a^	1.15^a^	0.25^a^	0^a^	0.92^a^	0.20^a^	0^a^	0^a^	0.88^a^	0^a^	1.16^a^	0.52^a^	1.44	1.67	–	–
**G3**	1.62^a^	2.47^a^	1.34^a^	0.49^a^	3.86^a^	0.98^a^	0.25^a^	0.1^a^	–	–	0^a^	0^a^	1.48^a^	0^a^	1.16^a^	1.04^a^	0.37	0.94	0.84^a^	0.30^a^
**G4**	2.11^a^	2.47^a^	2.39^a^	0.40^a^	0.68^a^	0.53^a^	0.17^a^	0^a^	–	–	0^a^	0^a^	0.89^a^	1.50^a^	0^a^	1.47^a^	3.12	1.60	1.18^a^	1.67^a^
**G5**	2.80^a^	2.47^a^	1.34^a^	3.40^a^	1.62^a^	0.75^a^	0.17^a^	0.23^a^	–	–	0^a^	0^a^	0.88^a^	0.76^a^	1.16^a^	0.52^a^	1.41	2	0.82^a^	0.08^a^
**G6**	0.93^a^	3.64^a^	1.13^a^	1.46^a^	2.37^a^	5.40^a^	0.17^a^	0.10^a^	–	–	0^a^	0^a^	–	–	–	–	1.04	2.8	1.49^a^	1.09^a^
**G7**	0.69^a^	1.28^a^	0.92^a^	0.32^a^	2.82^a^	4.18^a^	–	–	–	–	–	–	–	–	–	–	1.04	1.53	1.82^a^	1.55^a^
**G8**	0.93^a^	0.22^a^	2.09^a^	0.49^a^	3.90^a^	1.71^a^	0.17^a^	0.10^a^	–	–	0^a^	0^a^	–	–	2.33^a^	0.86^a^	1.37	1.53	1.21^a^	1.50^a^
**G9**	2.06^a^	0.12^a^	1.80^a^	0.49^a^	1.37^a^	1.46^a^	–	–	–	–	–	–	–	–	1.16^a^	2.36^a^	1.37	1.53	0.82^a^	0.87^a^
**G10**	1.62^a^	1.40^a^	2.26^a^	1.77^a^	1.53^a^	0.82^a^	0.57^a^	0.45^a^	–	–	0^a^	0^a^	0^a^	0.54^a^	2.33^a^	1.67^a^	1.37	1.06	0.65^a^	0.81^a^
**G11**	2.81^a^	1.98^a^	1.43^a^	0.49^a^	4.28^a^	2.52^a^	0.59^a^	0.45^a^	–	–	0^a^	2.27^a^	0.9^a^	0.59^a^	0^a^	0.39^a^	1.3	0.9	0.59^a^	0.41^a^
**G12**	1.62^a^	1.28^a^	1.82^a^	1.46^a^	2.72^a^	2.43^a^	–	–	–	–	–	–	–	–	2.33^a^	2.99^a^	–	–	–	–
***Mean***	***1.6***	***1.7***	***1.5***	***1.0***	***2.3***	***2.0***	***0.3***	***0.2***	***0.6***	***0.5***	***0.1***	***0.5***	***0.8***	***0.6***	***1.4***	***1.5***	***1.3***	***1.6***	***1.1***	***1.0***
																				
***SFBC specifications***	***4.0***	***4.0***	***8.0***	***8.0***	***8.0***	***8.0***	***2.0***	***1.8***	***2.5***	***2.5***	***3.5***	***3.5***	***2.3***	***2.3***	***10.0***	***10.0***	–	–	***10.0***	***10.0***

[H^+^]: proton concentration, *p*CO_2_: partial pressure of CO_2_, *p*O_2_: partial pressure of O_2_, Na^+^: sodium, K^+^: potassium, Cl^-^: chloride, Ca^2+^: calcium, Hb: hemoglobin, O_2_Hb: oxyhemoglobin.

a Result meets SFBC specifications [21].

### 3.4 Uncertainty in measurement

The results obtained for the determination of [H^+^], Cl^-^, K^+^, lactate, Ca^2+^, pCO_2_ and Hb met Ricos’ requirements (Table 5). However, for Na^+^, we observed that only four values obtained met Ricos’ requirements: level A for analyzer G4; levels A and B for G6 and level A for analyzer G8 (Table 5). For [H^+^], we observed only one value outside Ricos’ requirements: level B for analyzer G6 (Table 5).

**Table 5:** Uncertainty in measurement study on the 12 GEM PREMIER 4000 analyzers (G1–G12) at two levels. The results are presented in concentrations and as percentages. For [H^+^], *p*CO_2_, *p*O_2_, Na^+^, Cl^-^, K^+^ and Ca^2+^, assays were carried out on solutions A and B; for lactate, Hb and O_2_Hb, on solutions A and D.

	**[H**^**+**^**]** nmol/L (%)		***p*****CO**_**2**_ mmHg (%)		***p*****O**_**2**_ mmHg (%)		**Na**^**+**^ mmol/L (%)		**Cl**^**-**^ mmol/L (%)		**K**^**+**^ mmol/L (%)		**Ca**^**2+**^ mmol/L (%)		**Lactate** mmol/L (%)		**Hb** g/dL (%)		**O**_**2**_**Hb** % (%)	
	**Sol A**	**Sol B**	**Sol A**	**Sol B**	**Sol A**	**Sol B**	**Sol A**	**Sol B**	**Sol A**	**Sol B**	**Sol A**	**Sol B**	**Sol A**	**Sol B**	**Sol A**	**Sol D**	**Sol A**	**Sol D**	**Sol A**	**Sol D**
**G1**	1.40 (1.14^a^)	1.28 (3.28^a^)	1.64 (2.49^a^)	1.91 (5.60^a^)	3.96 (3.54)	9.45 (5.19)	1.3 (1.2)	2.3 (1.5)	0.8 (1.6^a^)	2.6 (3)	0.16 (2.31^a^)	0.17 (8.79)	0.03 (1.55^a^)	0.02 (3.04^a^)	0.33 (9.85^a^)	0.35 (4.29^a^)	0.31 (2.16^a^)	0.22 (3.03^a^)	0.99 (1.11)	1.99 (2.86)
**G2**	2.43 (1.97^a^)	1.53 (3.94^a^)	1.61 (2.44^a^)	1.89 (5.56^a^)	3.27 (2.95)	9.27 (5.15)	1.3 (1.3)	1.9 (1.3)	1.2 (2.5)	1.6 (1.8^a^)	0.10 (1.42^a^)	0.01 (0.4^a^)	0.05 (2.86^a^)	0.02 (2^a^)	0.19 (5.84^a^)	0.41 (5.15^a^)	0.25 (1.73^a^)	0.22 (3.07^a^)	–	–
**G3**	2.15 (1.75^a^)	1.51 (3.87^a^)	1.99 (2.98^a^)	1.91 (5.62^a^)	5.43 (4.84)	6.51 (3.60)	1.2 (1.2)	2.5 (1.6)	–	–	0.11 (1.50^a^)	0.01 (0.7^a^)	0.04 (2.09^a^)	0.02 (2^a^)	0.18 (5.51^a^)	0.37 (4.44^a^)	0.19 (1.30^a^)	0.31 (4.14^a^)	1.27 (1.42)	0.37 (0.53)
**G4**	4.52 (3.67^a^)	1.27 (3.28^a^)	2.44 (3.76^a^)	1.92 (5.64^a^)	3.32 (2.96)	4.96 (2.75)	0.9 (0.8^a^)	2 (1.3)	–	–	0.06 (0.86^a^)	0.08 (4.14^a^)	0.03 (1.64^a^)	0.02 (2.66^a^)	0.19 (5.83^a^)	0.38 (4.72^a^)	0.65 (4.48^a^)	0.23 (3.12^a^)	1.83 (2.04)	1.31 (1.88)
**G5**	3.56 (2.88^a^)	1.34 (3.45^a^)	1.93 (2.92^a^)	2.10 (6.17)	4.53 (4.08)	5.30 (2.94)	1.3 (1.2)	2.6 (1.7)	–	–	0.10 (1.42^a^)	0.01 (0.6^a^)	0.05 (2.90^a^)	0.02 (2.75^a^)	0.24 (7.23^a^)	0.47 (5.91^a^)	0.58 (4^a^)	0.44 (5.96^a^)	0.66 (0.74)	0.39 (0.57)
**G6**	2.37 (1.93^a^)	1.70 (4.37)	1.59 (2.45^a^)	1.92 (5.64^a^)	7.69 (6.93)	13.34 (7.41)	1.2 (1.1^a^)	1.7 (1.1^a^)	–	–	0.07 (0.96^a^)	0.07 (3.46^a^)	–	–	–	–	0.45 (3.14^a^)	0.14 (1.84^a^)	1.50 (1.68)	1.13 (1.63)
**G7**	1.68 (1.37^a^)	1.17 (3.00^a^)	2.38 (3.77^a^)	1.60 (4.69^a^)	5.64 (5.04)	10.51 (5.84)	–	–	–	–	–	–	–	–	–	–	0.24 (1.69^a^)	0.23 (3.04^a^)	1.73 (1.94)	1.53 (2.20)
**G8**	2.31 (1.88^a^)	1.29 (3.32^a^)	1.95 (2.95^a^)	1.94 (5.70^a^)	7.53 (6.78)	6.79 (3.73)	0.8 (0.8^a^)	1.9 (1.3)	–	–	0.10 (1.38^a^)	0.01 (0.60^a^)	–	–	0.33 (9.88^a^)	0.18 (2.25^a^)	0.39 (2.68^a^)	0.22 (3.03^a^)	1.16 (1.29)	1.48 (2.14)
**G9**	4.27 (3.47^a^)	1.02 (2.61^a^)	1.78 (2.74^a^)	1.64 (4.83^a^)	4.58 (4.13)	8.34 (4.63)	–	–	–	–	–	–	–	–	0.19 (5.83^a^)	0.94 (11.57^a^)	0.58 (4^a^)	0.23 (3.14^a^)	1.17 (1.31)	0.38 (0.55)
**G10**	2.87 (2.32^a^)	1.31 (3.35^a^)	1.98 (3^a^)	1.95 (5.70^a^)	4.61 (4.11)	6.28 (3.49)	1.7 (1.6)	2.9 (1.9)	–	–	0.11 (1.49^a^)	0.01 (0.4^a^)	0.02 (1.37^a^)	0.02 (3.04^a^)	0.17 (4.99^a^)	0.63 (7.75^a^)	0.59 (4.06^a^)	0.12 (1.55^a^)	1.18 (1.32)	0.96 (1.38)
**G11**	3.99 (3.25^a^)	1.32 (3.38^a^)	3.19 (4.91^a^)	1.73 (5.10^a^)	6.62 (5.96)	13.67 (7.60)	1.6 (1.5)	2.4 (1.6)	–	–	0.05 (0.74^a^)	0.14 (7.50^a^)	0.04 (2^a^)	0.02 (2.5^a^)	0.12 (3.60^a^)	0.30 (3.73^a^)	0.25 (1.72^aa^)	0.13 (1.80^a^)	0.81 (0.90)	0.67 (0.96)
**G12**	3.25 (2.60^a^)	1.33 (3.42^a^)	2.04 (3.09^a^)	1.85 (5.44^a^)	4.29 (3.86)	7.58 (4.19)	–	–	–	–	–	–	–	–	0.17 (4.99^a^)	0.88 (10.9^a^)	–	–	–	–
***Me**a**n***	***2.90 (2.35)***	***1.34 (3.44)***	***2.04 (3.13)***	***1.86 (5.47)***	***5.13 (4.60)***	***8.5 (4.71)***	***1,3 (1.2)***	***2,2 (1.5)***	***1 (2.1)***	***2.1 (2.4)***	***0,10 (1.34)***	***0,06 (3)***	***0,04 (2.06)***	***0,02 (2.57)***	***0,21 (6.36)***	***0,49 (6.07)***	***0,41 (2.81)***	***0,23 (3.07)***	***1.23 (1.38)***	***1.02 (1.47)***
										
***Ricos et** a**l. specific**a**tions***	***(3.9)***		***(5.7)***		–		***(1.1)***		***(2.2)***		***(8.4)***		***(3.1)***		***(15.2)***		***(6.3)***		–	

[H^+^]: proton concentration, *p*CO_2_: partial pressure of CO_2_, *p*O_2_: partial pressure of O_2_, Na^+^: sodium, K^+^: potassium, Cl^-^: chloride, Ca^2+^: calcium, Hb: hemoglobin, O_2_Hb: oxyhemoglobin.

a Result meets desirable specifications of Ricos et al. [22].

### 3.5 Methods comparison

These tests were carried out over the measurement ranges 6.80–7.58 for pH (100–22 nmol/L H^+^), 26–150 mmHg for *p*CO_2_, 33–203 mmHg for *p*O_2_, 125–163 mmol/L for Na^+^, 86–136 mmol/L for Cl^-^, 2.4–6.7 mmol/L for K^+^, 0.25–1.51 mmol/L for Ca^2+^, 0.8–19 mmol/L for lactate, 6.0–20.7 g/dL for Hb and 37.2–98.4% for O_2_Hb. The data obtained for pH, *p*O_2_, *p*CO_2_, Cl^-^, K^+^, Ca^2+^ and O_2_Hb were conformant. The slopes of the allometric plots obtained were close to 1, with intercept close to 0. In addition, the means of differences were close to 0 with relatively low standard deviations. The means of ratios were close to 1, still with low standard deviations (Table 6). For lactate, the data was also conformant for comparisons between GEM PREMIER 4000 analyzers (Table 6). For the comparison between GEM PREMIER 4000 G1 and the central laboratory, only the results exploited with a single range of values are presented (Table 6). The analysis of the results over two measurement ranges (0.8–3.8 mmol/L and 3.8–19 mmol/L) revealed a mean of differences close to 0 for the first range and a slight overestimation by Vista relative to G1 for the second range. For Na^+^ and Hb, the data was also conformant for all the comparisons (Table 6). We note two slopes greater than 1.1 for comparisons G3 *vs*. G1 on Na^+^ and G2 vs. G1 on Hb, but these are compensated by the intercepts. We note that on the graphs plotted, nearly all the points lay in the interval [− 2 SD; +2 SD]. In addition, the few exceptions did not change the clinical and biological interpretation of the results. Graphs were presented for comparisons between the central laboratory and G1 (Supplementary Fig. 1), and between 2 GEM PREMIER 4000 analyzers (G1, G3) (Supplementary Fig. 2). The method comparison between the 12 GEM PREMIER 4000 analyzers made on 43 GEM System Evaluator vials (5 level 1, 19 level 2 and 19 level 3), all from the same batch, run on all 12 analyzers showed no statistically significant differences between the analyzers for any of the parameters (Table 7).

**Table 6:** Method comparisons between 12 GEM PREMIER 4000 analyzers (G1–G12) and between GEM PREMIER 4000 analyzers and those of the central laboratory (Lab). The comparisons between the GEM PREMIER 4000 analyzers were made on 30 samples. The comparisons between G1 and the central laboratory were made on 50 samples for Na^+^ and Cl^-^, 40 samples for K^+^ and lactate, and lastly on 30 samples for Ca^2+^.

	**pH**			***p*****CO**_**2**_			***p*****O**_**2**_			**Na**^**+**^			**Cl**^**-**^		
	LRE (*a;b*)	*m*_d_ (σ)	*m*_r_ (σ)	LRE (*a;b*)	*m*_d_ (σ)	*m*_r_ (σ)	LRE (*a;b*)	*m*_d_ (σ)	*m*_r_ (σ)	LRE (*a;b*)	*m*_d_ (σ)	*m*_r_ (σ)	LRE (*a;b*)	*m*_d_ (σ)	*m*_r_ (σ)
**G1 vs. Lab**	–	–	–	–	–	–	–	–	–	0.98; 1.31	0.98 (1.545)	1 (0.011)	1.04; − 3.82	− 0.54 (1.446)	1 (0.014)
**G2 vs. G1**	1; − 0.07	0 (0.013)	1 (0.002)	1; 0.73	− 0.07 (1.363)	1 (0.022)	1; − 0.03	− 0.70 (3.042)	1 (0.048)	1.02; − 3.08	0.1 (0.885)	1 (0.006)	0.98; 2.65	− 0.27 (1.112)	1 (0.011)
**G3 vs. G1**	1; − 0.15	− 0.01 (0.009)	1 (0.001)	1; 0.78	0.6 (1.850)	1 (0.028)	1; − 0.87	0.47 (2.897)	1 (0.035)	1.12; − 18.36	1.1 (1.185)	1 (0.009)	–	–	–
**G4 vs. G1**	1; − 0.29	− 0.01 (0.015)	1 (0.002)	1.1; − 3.28	− 1.13 (3.730)	1 (0.047)	1; 0.88	− 0.47 (4.313)	1 (0.078)	1.05; − 8.54	1.47 (1.306)	1 (0.009)	–	–	–
**G5 vs. G1**	1; 0.08	0 (0.013)	1 (0.002)	1; 1.20	0.73 (1.999)	1 (0.033)	1; − 0.97	1.47 (3.655)	1 (0.032)	1.01; − 2.57	0.87 (1.502)	1 (0.011)	–	–	–
**G6 vs. G1**	1; 0.02	0 (0.014)	1 (0.002)	1; − 0.37	− 0.47 (2.713)	1 (0.037)	1; − 1.48	3.3 (3.650)	1 (0.036)	1.03; − 3.55	0 (1.050)	1 (0.007)	–	–	–
**G7 vs. G1**	1; 0.14	− 0.02 (0.010)	1 (0.001)	0.9; 2.18	1.9 (2.537)	1 (0.040)	1; − 2.36	0.77 (2.622)	1 (0.031)	–	–	–	–	–	–
**G8 vs. G1**	1; 0.16	0 (0.012)	1 (0.002)	1; − 0.24	− 0.47 (2.662)	1 (0.035)	1; − 0.63	− 0.3 (4.268)	1 (0.044)	0.98; 2.33	0.57 (0.728)	1 (0.005)	–	–	–
**G9 vs. G1**	1; − 0.02	− 0.01; (0.017)	1 (0.002)	1; − 1.04	0.23 (1.675)	1 (0.044)	1; 0.96	0.61 (3.625)	1 (0.030)	–	–	–	–	–	–
**G10 vs. G1**	1; 0.03	− 0.02 (0.009)	1 (0.001)	1; − 2.45	0.83 (1.984)	1 (0.032)	1; 3.73	− 1.2 (3.566)	1 (0.032)	1.01; − 2.90	1.73 (1.112)	1 (0.008)	–	–	–
**G11 vs. G1**	1; 0	− 0.01 (0.008)	1 (0.001)	1; − 1.69	1.43 (1.888)	1 (0.031)	1; − 1.44	1.17 (2.534)	1 (0.030)	1.02; − 4.58	2.3 (0.794)	1 (0.006)	–	–	–
**G12 vs. G11**	1; − 0.05	0 (0.010)	1 (0.001)	1; 2.59	− 0.37 (2.834)	1 (0.033)	1; − 2.68	1.37 (2.327)	1 (0.034)	–	–	–	–	–	–

	**K**^**+**^			**Ca**^**2+**^			**Lactate**			**Hb**			**O**_**2**_**Hb**		
	LRE (*a;b*)	*m*_d_ (σ)	*m*_r_ (σ)	LRE (*a;b*)	*m*_d_ (σ)	*m*_r_ (σ)	LRE (*a;b*)	*m*_d_ (σ)	*m*_r_ (σ)	LRE (*a;b*)	*m*_d_ (σ)	*m*_r_ (σ)	LRE (*a;b*)	*m*_d_ (σ)	*m*_r_ (σ)
**G1 vs. Lab**	1; − 0.17	0 (0.076)	1 (0.019)	0.9; 0.05	0.05 (0.045)	1 (0.057)	0.8; 0.20	0.29 (0.779)	1 (0.226)	1; − 0.1	− 0.20 (0.663)	1 (0.064)	–	–	–
**G2 vs. G1**	1; − 0.13	0 (0.069)	1 (0.018)	1; 0	0 (0.013)	1 (0.015)	1; − 0.06	− 0.04 (0.177)	1 (0.047)	1.3; − 3.03	− 0.14 (1.006)	1 (0.084)	–	–	–
**G3 vs. G1**	1; 0	0 (0.053)	1 (0.012)	1; 0	0.01 (0.015)	1 (0.015)	0.9; 0.46	0.25 (0.693)	1 (0.063)	1; − 0.25	0 (0.175)	1 (0.016)	0.9; 5.84	− 1.29 (0.783)	1 (0.01)
**G4 vs. G1**	1; 0	0 (0.064)	1 (0.017)	1; 0	0.01 (0.019)	1 (0.02)	1; 0.01	− 0.15 (0.193)	1 (0.033)	1; − 0.27	0.02 (0.376)	1 (0.032)	1; 1.89	− 1.03 (3.077)	1 (0.038)
**G5 vs. G1**	1; 0.01	− 0.03 (0.061)	1 (0.016)	1; 0	− 0.01 (0.022)	1 (0.023)	1; − 0.09	0.11 (0.181)	1 (0.045)	1; − 0.42	0.08 (0.227)	1 (0.020)	1.1; − 8.09	− 0.94 (1.214)	1 (0.014)
**G6 vs. G1**	1; 0	0 (0.059)	1 (0.014)	–	–	–	–	–	–	1; − 0.1	0 (0.211)	1 (0.020)	1; − 1.29	− 0.68 (0.748)	1 (0.008)
**G7 vs. G1**	–	–	–	–	–	–	–	–	–	1; − 0.13	− 0.03 (0.151)	1 (0.014)	1; 0.23	− 1.05 (0.466)	1 (0.005)
**G8 vs. G1**	1; 0.19	− 0.04 (0.077)	1 (0.019)	–	–	–	1; 0.06	0.01 (0.225)	1 (0.047)	1.1; − 0.90	− 0.03 (0.415)	1 (0.038)	1; − 0.39	− 0.68 (1.171)	1 (0.015)
**G9 vs. G1**	–	–	–	–	–	–	0.9; 0.35	− 0.04 (0.425)	1 (0.073)	1; − 0.05	0.11 (0.244)	1 (0.025)	0.95; 5.64	− 1.20 (0.711)	1 (0.008)
**G10 vs. G1**	1; 0.1	0.01 (0.052)	1 (0.013)	1; 0.02	0.01 (0.023)	1 (0.022)	1; − 0.07	− 0.12 (0.174	1 (0.028)	1; 0.10	0.33 (0.297)	1 (0.035)	1; 3.03	− 1.71 (0.818)	1 (0.01)
**G11 vs. G1**	1; 0.05	0.01 (0.061)	1 (0.016)	1; 0	0.02 (0.015)	1 (0.016)	1; − 0.22	0 (0.167)	1 (0.025)	1; − 0.26	− 0.02 (0.157)	1 (0.014)	1; 4.57	− 0.97 (0.507)	1 (0.007)
**G12 vs. G11**	–	–	–	–	–	–	1; 0	0.13 (0.144)	1 (0;027)	–	–	–	–	–	–

LRE: Least rectangles equation. pH: hydrogen potential, *p*CO_2_: partial pressure of CO_2_, *p*O_2_: partial pressure of O_2_, Na^+^: sodium, K^+^: potassium, Cl^-^: chloride, Ca^2+^: calcium, Hb: hemoglobin, O_2_Hb: oxyhemoglobin. *a*: slope of allometric plot, *b*: intercept of allometric plot, *m*_d_: mean of differences, *m*_r_: mean of ratios, σ: standard deviation.

**Table 7:** Method comparison between 12 GEM PREMIER 4000 analyzers (G1–G12). The comparisons between the GEM PREMIER 4000 analyzers were made on 43 GEM System Evaluator vials (5 of level 1, 19 of level 2 and 19 of level 3), all from the same batch, run on all 12 analyzers. The comparisons between the means obtained were made with an ANOVA test.

	**G1**	**G2**	**G3**	**G4**	**G5**	**G6**	**G7**	**G8**	**G9**	**G10**	**G11**	**G12**	***ANOVA***
**pH:*****m*****(σ)**	7.44 (0.14)	7.44 (0.14)	7.44 (0.14)	7.44 (0.14)	7.44 (0.14)	7.44 (0.14)	7.44 (0.14)	7.44 (0.14)	7.44 (0.14)	7.44 (0.14)	7.44 (0.14)	7.44 (0.14)	***NS***
***p*****CO**_**2**_**:*****m*****(σ)**	31.2 (21.3)	31.5 (21.1)	31.4 (20.8)	31.4 (20.4)	31.3 (19.9)	31.2 (21.5)	31.2 (21.1)	31.6 (20.5)	31.8 (21.4)	31.4 (21)	31 (21.1)	31.4 (20.6)	***NS***
***p*****O**_**2**_**:*****m*****(σ)**	199 (146)	200 (143)	201 (151)	200 (148)	201 (144)	203 (149)	202 (150)	199 (144)	188 (148)	204 (152)	203 (152)	196 (146)	***NS***
**Na**^**+**^**:*****m*****(σ)**	145 (11)	145 (10)	145 (11)	145 (10)	145 (11)	145 (11)	–	146 (11)	–	144 (11)	145 (11)	–	***NS***
**K**^**+**^**:*****m*****(σ)**	5.6 (1.8)	5.6 (1.8)	5.7 (1.9)	5.6 (1.8)	5.7 (1.9)	5.7 (1.9)	–	5.6 (1.8)	–	5.7 (1.9)	5.7 (1.9)	–	***NS***
**Ca**^**2+:**^***m*****(σ)**	0.99 (0.31)	0.99 (0.31)	0.98 (0.31)	0.99 (0.31)	0.99 (0.31)	–	–	–	–	0.97 (0.32)	0.98 (0.32)	–	***NS***
**Lactate:*****m*****(σ)**	2.20 (1.91)	2.16 (1.92)	2.21 (1.93)	2.25 (2.04)	2.19 (1.92)	–	–	2.24 (1.92)	2.22 (1.90)	2.30 (2.03)	2.28 (2.00)	2.28 (2.00)	***NS***
**Hb:*****m*****(σ)**	12.02 (4.46)	12.03 (4.49)	11.96 (4.45)	11.96 (4.47)	12.08 (4.53)	11.9 (4.43)	12.04 (4.5)	12.11 (4.58)	12.05 (4.53)	11.95 (4.46)	11.88 (4.44)	–	***NS***
**O**_**2**_**Hb:*****m*****(σ)**	77.96 (17.5)	–	77.95 (17.49)	77.96 (17.5)	77.96 (17.5)	77.95 (17.5)	77.95 (17.49)	77.95 (17.49)	77.96 (17.5)	77.96 (17.5)	77.95 (17.49)	–	***NS***

pH: hydrogen potential, *p*CO_2_: partial pressure of CO_2_, *p*O_2_: partial pressure of O_2_, Na^+^: sodium, K^+^: potassium, Cl^-^: chloride, Ca^2+^: calcium, Hb: hemoglobin, O_2_Hb: oxyhemoglobin.

*m*: mean, σ: standard deviation, NS: not significant.

## 4 Discussion

In the context of deploying 12 point-of-care GEM PREMIER 4000 biological analyzers in the Clermont-Ferrand university teaching hospital, we carried out an on-site method validation (precision and method comparison) to meet the requirements of the standards NF EN ISO 15189 and 22870 [1], [2], [3], [4], [5], [6]. We also evaluated uncertainty in measurement (calculated with an approach using both reproducibility and accuracy data) at 2 levels for each parameter. There is not much data available for some parameters such as pO_2_ and O_2_Hb.

The precision study (repeatability, reproducibility) was conducted at two control levels for all our measured parameters, i.e. [H^+^], *p*CO_2_, *p*O_2_, Na^+^, K^+^, Cl^-^, Ca^2+^, Hb and O_2_Hb. For [H^+^], *p*CO_2_, Na^+^, K^+^, Cl^-^, Ca^2+^ and O_2_Hb, all the coefficients of variation (CVs) we obtained complied with the SFBC recommendations [22]. As there were no SFBC recommendations for Hb, the CV values were compared and found to be compliant with the recommendations of Ricos et al. [22]. For *p*O_2_, CV values for reproducibility were compliant with SFBC recommendations [21]. By contrast, the CV values for repeatability were compliant only for 3 levels out of the 24 tested; the other CV values for *p*O_2_ conformed to the supplier's recommendations (4.6%). These supplier's recommendations were based upon guidelines published by the Clinical and Laboratory Standards Institute (Wayne, PA, USA) [23]. All the CV values were below 4%. In addition, in our external quality control surveys (ASQUALAB), no nonconformity was established for this parameter (and the others); since it is highly labile on contact with ambient air it is difficult to perform repeat determinations is. Indeed, the bias values obtained in accuracy study met SFBC requirements for [H^+^], *p*O_2_, *p*CO_2_, Na^+^, K^+^, Cl^-^, Ca^2+^ and lactate [21]. This being the case, the criteria of Vassault et al. seem difficult to meet [14], [15]. The fact that all these reproducibility values, calculated from controls tested automatically on solutions carried by iQM cartridges, fulfill SFBC requirements, validates the self-monitoring strategy managed by the iQM [13], [17], [18]. A quantitative statement of the uncertainty in measurement (UM) is required to help interpret patients’ results. The results obtained for the UM determination of all analytes met available Ricos requirements with the exception of Na^+^ and [H^+^]. For Na^+^, we observed all the analyzers were consistent with data published by Matar et al. [24]. For [H^+^], only one value was outside Ricos’ requirements, with no impact on patient's results. There are no recommendations (Ricos, literature data or other) for pO_2_ and O_2_Hb. In this study, we propose 24 values of UM (12 values at each of 2 levels calculated with an approach using both reproducibility and accuracy dat^a^) for the first time for these 2 parameters. Our values were conformed with the UM calculated by the long-term analytical CV method (using only external quality control material) [25] at 8.4% and 2.9% for pO_2_ and O_2_Hb respectively.

For method comparisons, because of the large number of analyzers in the Hospital, we opted to make comparisons using a transitive model. In this model, the BACKUP (G1) analyzer was considered as the reference GEM PREMIER 4000 instrument. It was first compared with the automated analyzers at the central laboratory (Vista®, RapidPoint® and XN®), and then with all the other GEM PREMIER 4000 analyzers. All our comparisons between GEM PREMIER 4000 analyzers proved conformant, in both analysis of graphs and processing of numerical data. The recent COFRAC recommendations prescribe simultaneous comparison using an ANOVA test of all the analyzers performing the same analysis in a laboratory. The large number of instruments in our study, the low stability of some parameters, such as *p*O_2_, and the geographical locations of our analyzers make it difficult to perform such a comparison with blood samples. We decided to compare all the analyzers with 516 quality control vials (43 per analyzer) at three different levels and from the same batch (no statistically significant differences).

The comparison between GEM PREMIER 4000 analyzers and automated central laboratory analyzers was made for the first time. For COFRAC, the analyses can be considered different according to whether they were performed at the central laboratory or at the point of care: neither the matrix (heparinized whole blood for POCT versus heparinized plasma or serum for the central laboratory) nor the analysis method were the same. A comparative study thus remains to be conducted so that hospital laboratory staff can confidently advise clinicians on the simultaneous interpretation of tests done at the central laboratory and at point of care. Overall, the comparisons were conformant. This is particularly important for some parameters such as blood sodium: variations in concentration can occur according to the assay method (direct or indirect potentiometry) [26]. With indirect potentiometry, the results of Na^+^ determinations can vary owing to high blood lipid or protein levels, which modify the plasma water content and underestimate the Na^+^ titer [26]. In addition, as emphasized when vetting users, preanalytical errors can cause discrepancies in results [14].

In conclusion, in view of the compliance of our data with standard recommendations, this work supports the use of 12 GEM PREMIER 4000 analyzers for POCT in services of academic medical centers, as exemplified by Clermont-Ferrand university teaching hospital.
