# Supplementary material for: Method validation of a set of 12 GEM® Premier™ 4000 blood gas analyzers for point-of-care testing in a university teaching hospital
Source: Pract Lab Med. 2017 Dec 13;10:21–33. doi: 10.1016/j.plabm.2017.12.001 (PMC5814368; doi:10.1016/j.plabm.2017.12.001)

SUPPLEMENTARY DATA LEGENDS

Supplementary, Figure 1. Comparison of results between central laboratory (Lab) and G1 with least rectangle regressions (A, B, C, D, E, F) and difference plots (G, H, I, J, K, L) for Na^+ (A, G), Cl^- (B, H), K^+ (C, I), Ca^{2+} (D, J), lactate (E, K), Hb (F, L).

Na^+: sodium, Cl^-: chloride, K^+: potassium, Ca^{2+}: calcium, Hb: hemoglobin.

Mean of differences   +/- 1.96 standard deviation

Supplementary, Figure 2. Comparison of results between G1 and G3 with least rectangle regressions (A, B, C, D, E, F, G, H, I) and difference plots (J, K, L, M, N, O, P, Q, R) for pH (A, J), pCO_2 (B, K), pO_2 (C, L), Na^+ (D, M), K^+ (E, N), Ca^{2+} (F, O), lactate (G, P), Hb (H, Q), O_2Hb (I, R).

pH: hydrogen potential, pCO_2: partial pressure of CO_2, pO_2: partial pressure of O_2, Na^+: sodium, K^+: potassium, Ca^{2+}: calcium, Hb: hemoglobin, O_2Hb: oxyhemoglobin.

Mean of differences   +/- 1.96 standard deviation

Supplementary, Figure 2 (Oris et al., 2017)

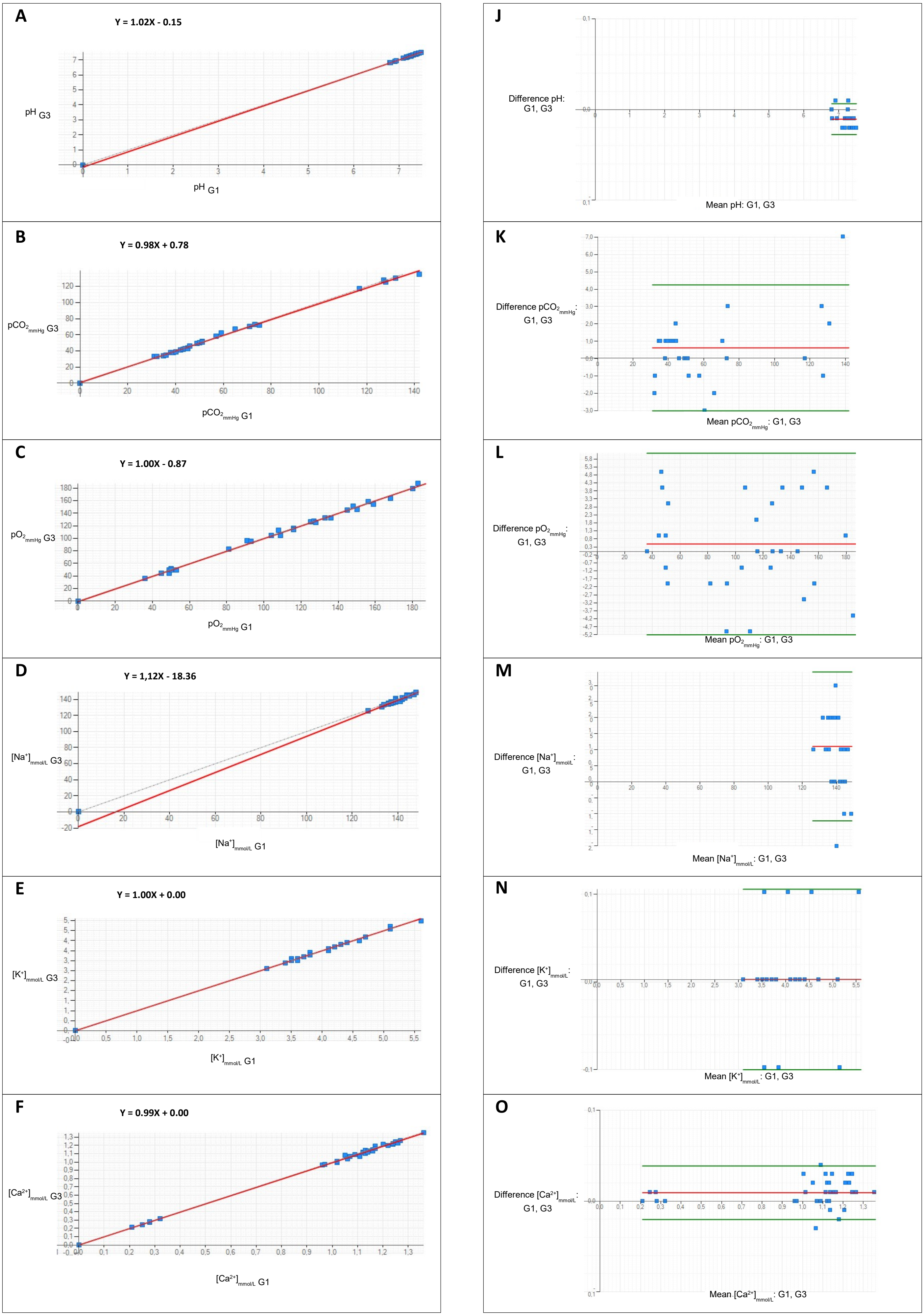

Supplementary, Figure 2 (Oris et al., 2017)

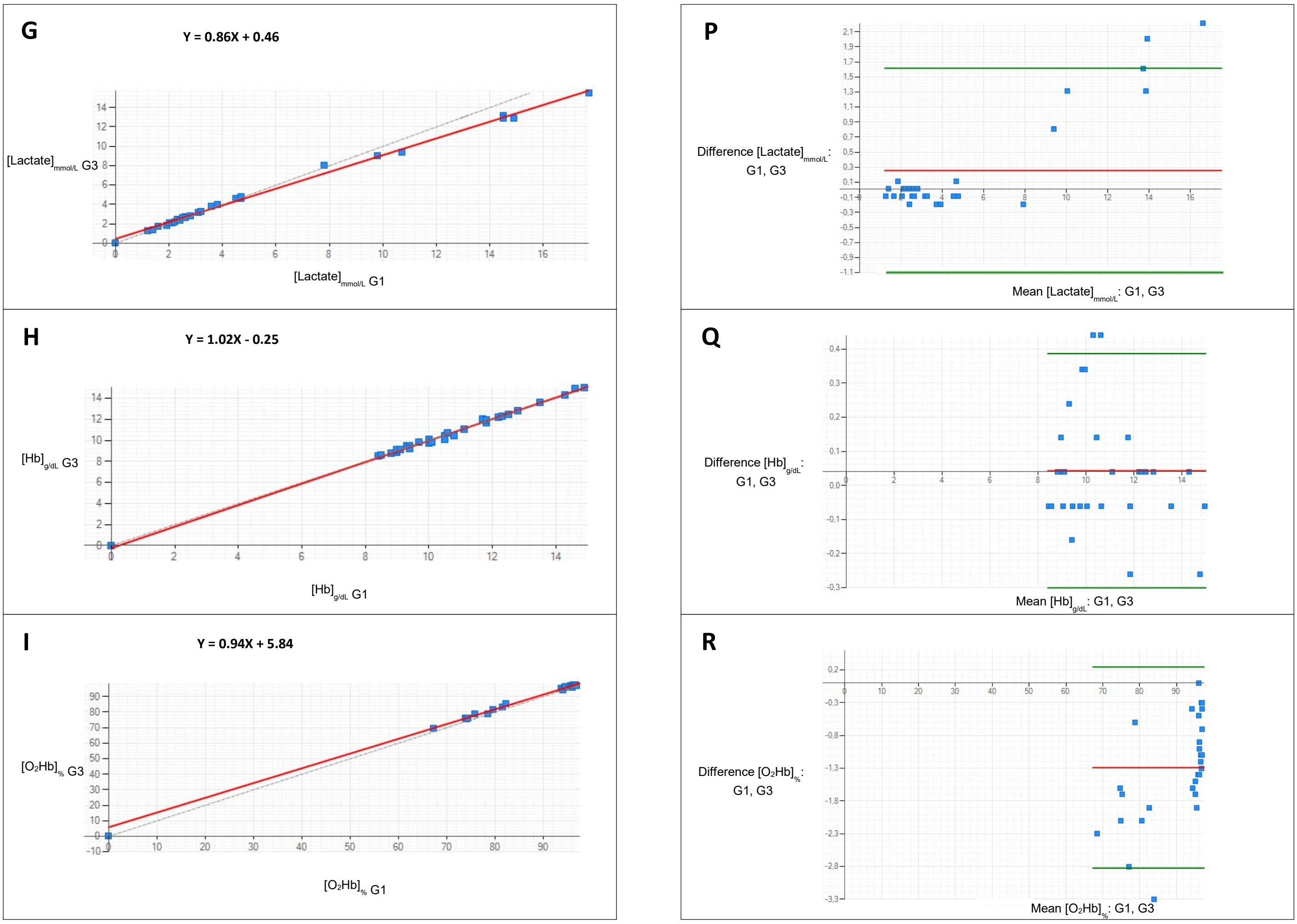

Supplementary , Figure 1 (Oris et al., 2017)

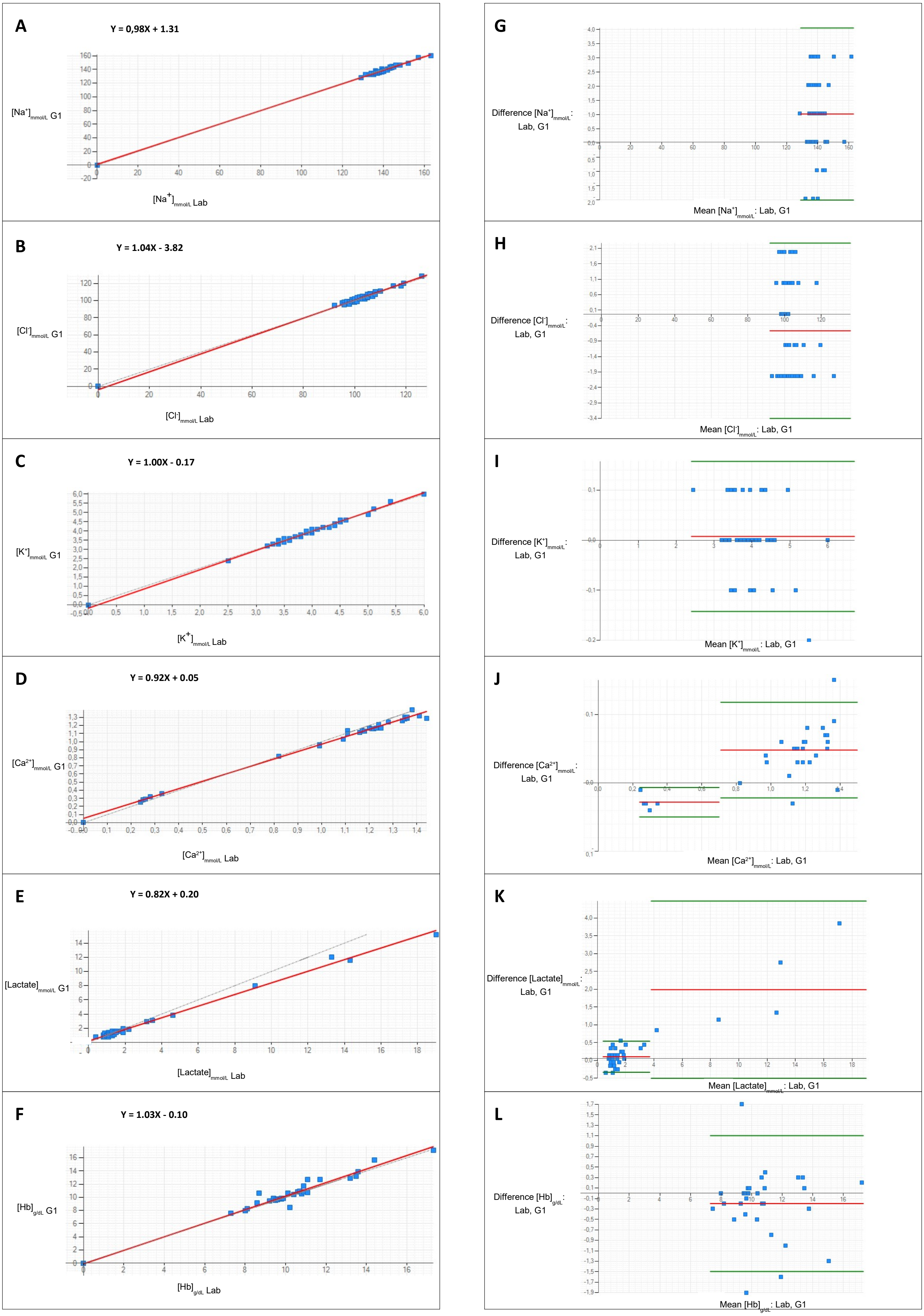

Supplement: Supplementary material [file mmc1.pdf]
